# Mitral Regurgitation Due to Caseous Calcification of the Mitral Annulus

**DOI:** 10.7759/cureus.11402

**Published:** 2020-11-09

**Authors:** Donovan Tran, Colin Zuchowski

**Affiliations:** 1 Diagnostic Radiology, University of Arizona College of Medicine, Tucson, USA; 2 Diagnostic Radiology, Emory University School of Medicine, Atlanta, USA

**Keywords:** caseoma, mitral regurgitation, caseous calcification of the mitral annulus

## Abstract

Mitral regurgitation can be a debilitating disease that has many etiologies. Frequent causes are mitral valve prolapse, rheumatic fever, dilated cardiomyopathy, and infective endocarditis. Another rare, but often overlooked cause is caseous calcification of the mitral annulus. This rare disease can lead to dangerous complications such as stroke and arrhythmias. In this report, we present an 84-year-old male with chronic kidney disease who was found to have mitral regurgitation secondary to caseous calcification of the mitral annulus. The goal of this report is to bring clinical awareness to this disease so that it is included in the differential diagnosis of mitral regurgitation.

## Introduction

Caseous calcification of the mitral annulus, also known as mitral annular caseoma, is a form of mitral annular calcification. This cardiac mass is often incidentally found on echocardiogram or computed tomography (CT) imaging. Reports of it being misdiagnosed as a cardiac tumor, abscess, or infective endocarditis have been reported [1]. The goal of this report is to bring clinical awareness of this disease to clinicians so that it is included in the differential diagnosis for patients presenting with mitral regurgitation.

## Case presentation

An 84-year-old male presented to the cardiothoracic surgery clinic to discuss options to treat his severe mitral regurgitation, as his dyspnea had slowly been worsening. He had a medical history of mitral regurgitation, chronic kidney disease, severe coronary artery disease requiring coronary artery bypass grafting, and heart failure with preserved ejection fraction of 65%.

Transesophageal echocardiogram revealed mitral valve leaflets that were diffusely thickened and degenerative with a flail secondary chord originating from the P2 scallop of the mitral valve. There was predominately A3-P3-P2 regurgitation that was the result of degenerative malcoaptation and flail secondary chord of the P2 scallop. The calculated effective regurgitant orifice area was 0.53 cm^2^ with a regurgitant volume of 99 mL; these values were indicative of severe mitral regurgitation. Additional findings were moderate aortic regurgitation with paravulvular leak, mild tricuspid regurgitation, left atrial dilation, and normal left ventricular function of 55%-60%.

**Figure 1:**
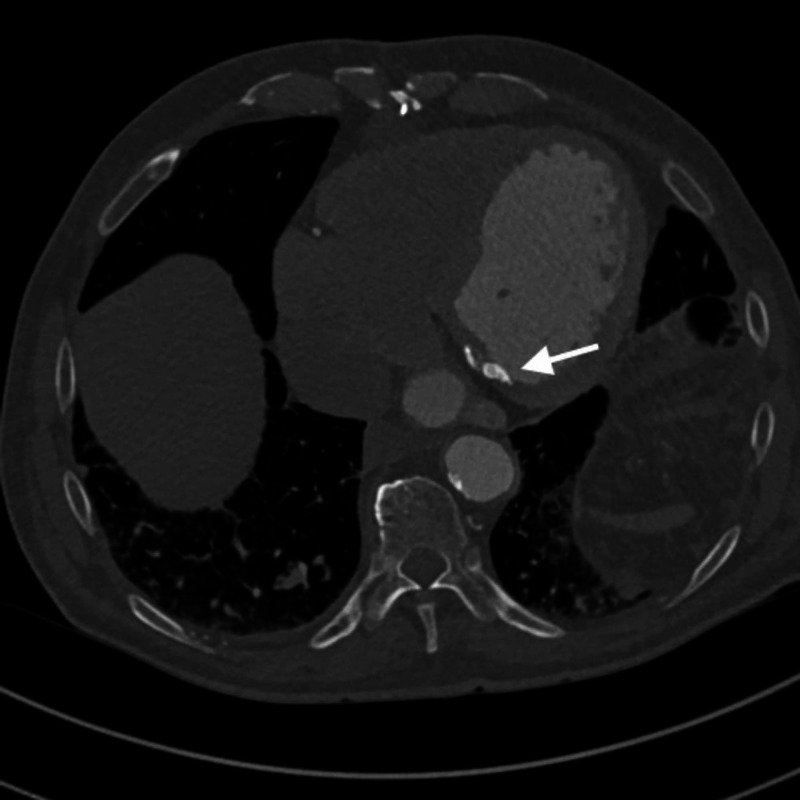
Axial contrast-enhanced coronary CT at the level of the mitral annulus demonstrates hyperdense calcific deposits consistent with annular calcifications.

**Figure 2:**
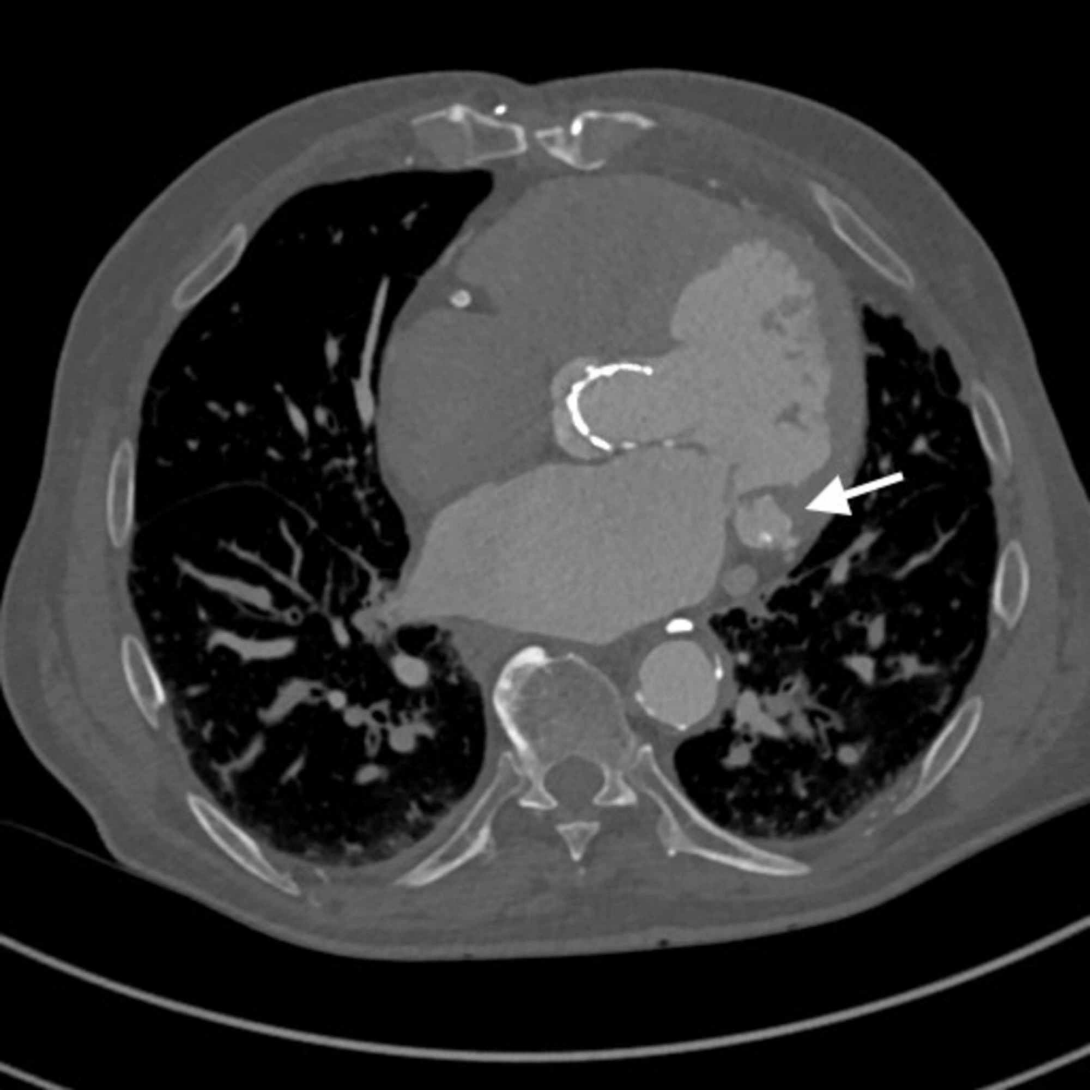
Axial contrast-enhanced coronary CT superiorly to Figure 1. Internal lower density material is seen, raising suspicion for internal necrosis.

**Figure 3:**
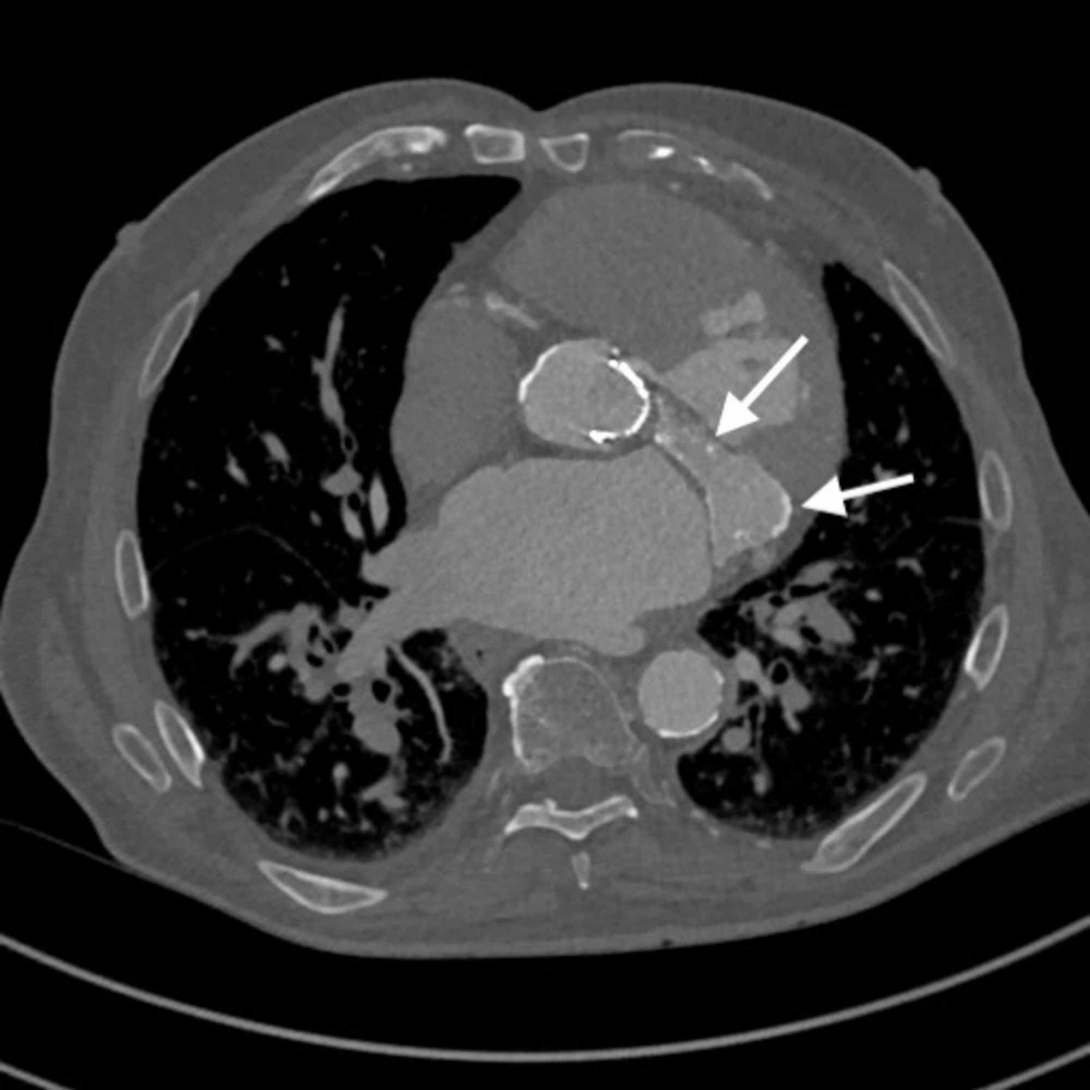
Axial contrast-enhanced CT superiorly to Figure 2. Frank enlargement and internal necrosis with circumferential calcification throughout the mitral valve annulus is better visualized.

Contrast-enhanced coronary CT showed global cardiomegaly with severe dilatation of the left atrium and eccentric posterior mitral annular calcification with a hypodense central region. These findings were highly suspicious for a caseoma (Figures 1-3).

## Discussion

Mitral annular caseomas, a rare variant of mitral annular calcification, is a chronic, degenerative process of the fibrous support structure of the mitral valve. Risk factors include increased age, mitral valve disease, cardiovascular disease, chronic kidney disease [2]. Mitral annular calcification was previously considered a benign finding but is now considered to be associated with a higher risk of cardiovascular mortality, atrial fibrillation, stroke, and mitral regurgitation [2]. Caseomas occur in 0.6% of patients with mitral annular calcification [3].

The pathogenesis of caseomas are not completely understood. It is thought to be related to the development of atherosclerosis due to many reports linking the two comorbidities. Other studies have also pointed to an association with altered calcium-phosphate metabolism due to its higher disease prevalence in patients with end-stage renal disease [4]. Our patient had chronic kidney disease and atherosclerosis; both these risk factors likely leading him to develop mitral annular caseomas.

Caseomas typically appears as a solid intramyocardial mass that adheres to the posterior portion of the mitral valve. Operative findings reported that grossly it appears as a “toothpaste-like, white, caseous material” [1]. Under microscopy, the caseous material has a central region of amorphous eosinophilic acellular material surround by macrophages and lymphocytes while the periphery has multiple calcifications [5].

Caseomas are often detected by transesophageal echocardiography or CT imaging. On echocardiogram, it appears as a hyperechoic mass with posterior acoustic shadowing on the mitral valve secondary to the calcification. By changing the acoustic window or viewing from a different angle as to reduce the posterior acoustic shadowing, internal central areas of hypoechogenicity can be seen, corresponding to the areas of necrosis [2]. Similarly, on CT imaging, the areas of calcific deposits along the mitral annulus will demonstrate areas of hyperdensity with the internal hypodensity corresponding to the areas of internal necrosis. On contrast-enhanced imaging, there will not be increased areas of enhancement seen.

Mitral annular calcification should not be confused with caseous necrosis due to Mycobacterium tuberculosis infection. Caseous necrosis often occurs in the lungs at the center of a granuloma, an entity not found in mitral annular calcification. Our patient had no history of tuberculosis.

Our patient presented with dyspnea due to mitral regurgitation. Mitral regurgitation has been reported in several cases of caseous calcification of the mitral annulus [6]. Other reported symptoms have included cerebral embolisms, mitral stenosis, cardiac conduction abnormalities, and spontaneous fistualization of the caseous material [7].

There is no standard treatment for caseomas. A small amount of calcium deposition in the mitral valve annulus is often clinically insignificant and is managed conservatively while severe cases are treated with mitral valve replacement via sternotomy [8]. Prior to surgery, a Society of Surgery (STS) risk score is often calculated and serves as a determinant of eligibility for cardiac procedures. The higher the STS risk score, the higher the mortality risks with mitral valve replacement via sternotomy. In this case, inserting a transcatheter mitral valve can sometimes be performed if the calcium deposition is not severe. 

The STS risk score for our patient was in the higher range. It was determined as 13.4% for death, 8.9% for permanent stroke, and 11.2% for renal failure with traditional sternotomy/mitral valve repair. Unfortunately, the patient was found to have excessive calcium deposition of his mitral valve and will be unable to have a transcatheter mitral valve repair. His only treatment option is to have a mitral repair via sternotomy which poses a high mortality risk. Currently, the patient is still in discussion with his medical team regarding whether to proceed with surgery. At the age of 85, thorough quality of life and goal of treatment discussions are required before pursuing such a high-risk procedure.

## Conclusions

Caseous calcification of the mitral annulus is a rare disease that is frequently overlooked. It is often misdiagnosed as a cardiac tumor, abscess, or endocarditis. This report aims to bring clinical awareness to this entity in patients with chronic kidney disease and atherosclerosis presenting with dyspnea.

## References

[1] Harpaz D, Auerbach I, Vered Z, Motro M, Tobar A, Rosenblatt S (2001). Caseous calcification of the mitral annulus: a neglected, unrecognized diagnosis. J Am Soc Echocardiogr.

[2] Abramowitz Y, Jilaihawi H, Chakravarty T, Mack MJ, Makkar RR (2015). Mitral annulus calcification. J Am Coll Cardiol.

[3] Alkadhi H, Leschka S, Prêtre R, Perren A, Marincek B, Wildermuth S (2005). Caseous calcification of the mitral annulus. J Thorac Cardiovasc Surg.

[4] Akram M, Hasanin AM (2012). Caseous mitral annular calcification: is it a benign condition?. J Saudi Heart Assoc.

[5] Elgendy I, Conti CR (2013). Caseous calcification of the mitral annulus: a review. Clin Cardiol.

[6] Marcì M, Jacono FL (2009). Mitral regurgitation due to caseous calcification of the mitral annulus: two case reports. Cases J.

[7] Gupta S, Ge Y, Ghouri M, Blankstein R, Steigner ML, Aghayev A (2018). Caseous calcification of the mitral annulus with atrial and ventricular fistulization. Circ Cardiovasc Imaging.

[8] Fong LS, Mclaughlin AJ, Okiwelu NL, Nordstrand IAJ, Newman M, Passage J, Joshi PV (2017). Surgical management of caseous calcification of the mitral annulus. Ann Thorac Surg.

